# Prevalence of Musculoskeletal, Neurological and Physical Disorder After COVID-19 in Saudi Arabia: A Cross Sectional Study

**DOI:** 10.12688/f1000research.109007.1

**Published:** 2022-04-26

**Authors:** Hayam Mahmoud, Anwar Ebid, Mohammed Alghamdi, Abeer Ibrahim, Ahmed Almoosa

**Affiliations:** 1 Physical Therapy, Umm-Al-Qura University, Makkah, 21955, Saudi Arabia; 2 Faculty of Physical Therapy, Cairo University, Cairo, Egypt; 3 Rehabilitation Department- KAMC, Ministry of National Guard- Health Affairs, Jeddah, Saudi Arabia

**Keywords:** COVID-19, headache, muscle weakness, respiratory difficulties, physical activities

## Abstract

Background: The global pandemic of SARS-CoV-2, or COVID-19 continues to attack all human systems. Although COVID-19 is a respiratory disease, various extra-pulmonary manifestations, including musculoskeletal and neuropathies/myopathies was reported. This study aimed to investigates the long-term impacts of COVID-19 infection on physical health, capability of daily life activities, musculoskeletal and neurological functions in the Kingdom of Saudi Arabia (KSA). Methods: A total of 499 adults recovered from COVID-19 infection of both sexes, who resided in the KSA were recruited randomly and invited to participate in this cross-sectional web-based survey. A self-administered structured questionnaire was used as an instrument of data collection. All respondents returned the questionnaire. Their responses were recorded, stored into a Microsoft Excel sheet 2010 and analyzed with the Statistical Package for the Social Sciences (SPSS) version 24. Percentages were used to convey descriptive data. The percentages were presented with a 95% confidence interval (CI). For statistical significance, a 0.05 p-value was used. Results: The overall prevalence of neurological and musculoskeletal disorders as follows: headache (63.1%), muscle ache or weakness (62.3%), vertigo (25%), concentration problems (21.8%), breathing troubles (20.4%), loss of balance (19.4%), seizure (1%), and Guillain-Barre Syndrome (0.6%). The results also revealed a significant association between the influence of COVID-19 infection and daily activities, gender and respiratory disorders. Conclusion: The findings highlighted and concluded that COVID-19 infection had an impact on respiratory, nervous, musculoskeletal systems and affect daily activities.

## Introduction

The global pandemic of coronavirus disease 2019, commonly known as COVID-19, continues to spread. The clinical presentations of COVID-19 varied from asymptomatic to severe critical condition. Although COVID-19 is a respiratory disease, numerous investigations documented extrapulmonary signs and symptoms. Musculoskeletal complaints, myopathies, and neuropathies are common post COVID-19 clinical presentations. ^1^ Patients with multiple manifestations ranged from asymptomatic or moderate (70% of cases) to severe or possibly fatal (30% of cases). ^2^ Although the severity of the symptoms varied with age and medical history of chronic diseases (comorbidity, such as chronic cardiac disease, lung disease, diabetes, chronic renal disease, and cancer). The most common symptoms included fever, cough, shortness of breath, fatigue, myalgia, and gastrointestinal symptoms. These symptoms may last for a long time even after recovery. ^3^^,^^4^ Symptoms might linger for weeks or months, and some people never return to their previous level of health. ^4^

In this regard, the emergence of COVID-19 has compelled governments worldwide to take different steps and decisions to prevent the pandemic from spreading quickly. ^5^ Social distancing, capacity constraints in public venues and private houses, isolation, quarantine, and curfew enforcement were among the preventative measures employed. ^6^ Saudi Arabia have been eager to apply social distancing and quarantine in order to avoid illness spread.

As a result, those preventative measures may have physical, emotional, and psychological impacts on people’s life. According to Mattioli *et al.*, precautionary measures had a negative impact on individuals, the society, and the economy which resulted in (a) an increase of community anxiety and tension, (b) an economic decline, (c) decreased outdoor exercises and overall physical activity (PA), (d) and development of both stress and depression, which can lead to unhealthy dietary habits. ^5^^,^^7^

Although there are numerous examples of long-term problems after COVID-19 infection, there is lack of research to support. There was a loss in patients’ functional capacity, which was correlated to the reduction in numbers of health-related quality-of-life indices. There was also evidence of cognitive problems, such as confusion and memory loss. High blood pressure, obesity, and mental health disorders were identified as risk factors for the persistence of these symptoms. ^6^ The importance of physical therapy in dealing with these issues should not be overstated. It is crucial to begin studying the long-term effects of COVID-19 infection. ^7^ To our knowledge, there is a lack of information and no previous study on the correlation between the prevalence of neurological, musculoskeletal, and physical disorders and COVID-19 in KSA. So, we aimed in this study to determine the long-term impacts of COVID-19 on physical health, daily life activities, musculoskeletal and neurological functions in the KSA.

## Methods

### Ethical considerations

This was an analytical cross-sectional survey study. The King Abdullah International Medical Research Center (KAIMRC) approved and considered this study ethically feasible (Approval study number: SP21J/015/02, E-CTS Ref. No. JED-21-427780-20935 and IBR NCBE Registration No.:H-01-R-005).

### Participants

A total number of 499 patients of both genders, aged from 20 to 60 years old who had issues following COIVD-19 and lived in Saudi Arabia were included in this study. All participants received full information and an explanation of the study’s objectives, advantages, and any potential hazards associated with participation. All participants signed an informed consent form indicating their willingness to participate in this study. A random sampling was performed based on city districts. This research was carried out between October 2020 and March 2021.

### Sampling

**Figure 1.**
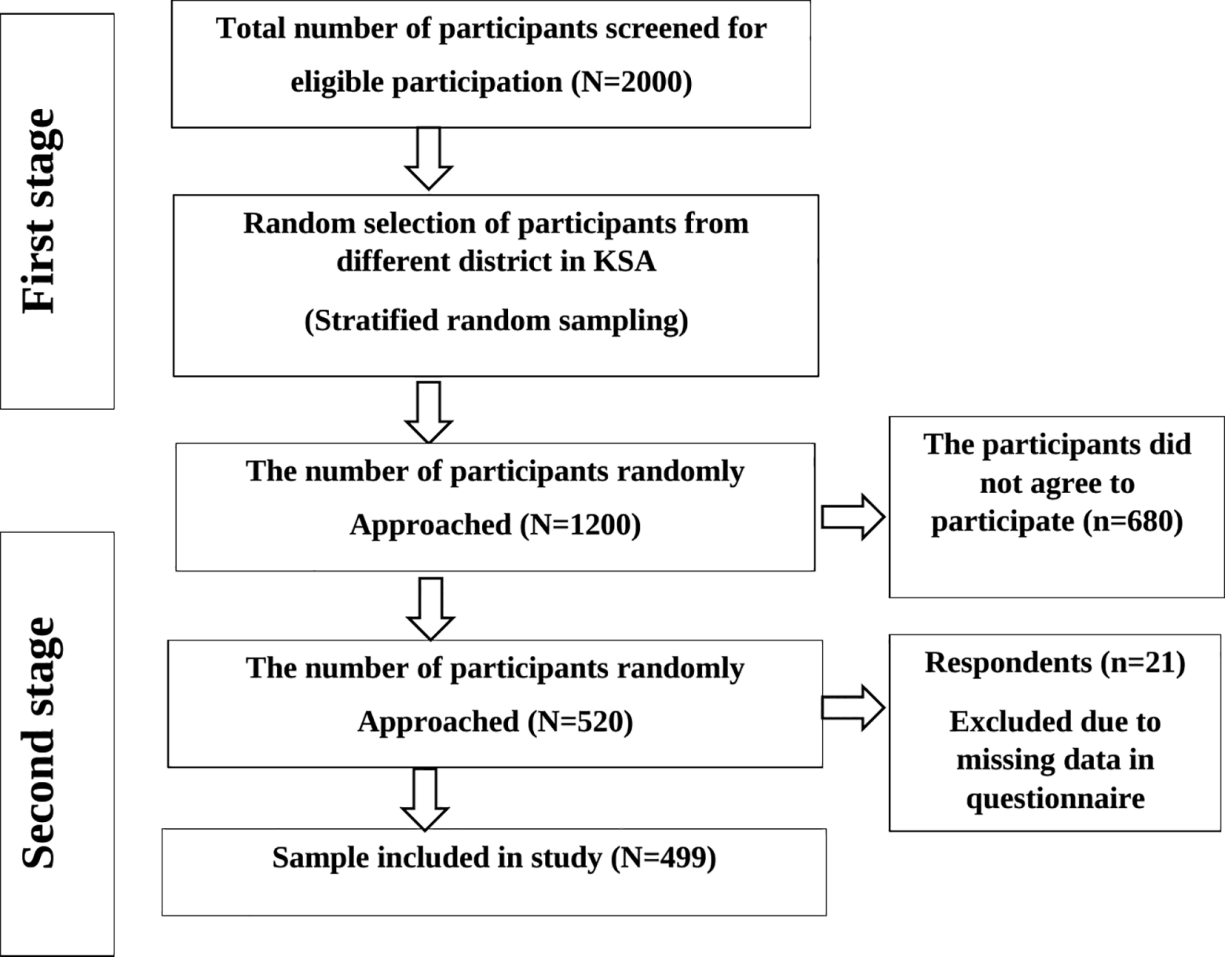
Flow diagram of sample selection.

The required sample size was calculated using Raosoft sample size calculator (Raosoft, Inc., Seattle, WA, USA), with a confidence interval of 99%, a response rate of 70%, and a margin error of 5%. The sample size was 1200 participants. A two-stage sampling approach was employed to select participants, with a random sampling of participants from different district followed by a random selection of participants from those districts. During the first sampling stage, random selection was conducted on a list of patients in the hospital database and 520 patients returned the questionnaire. 21 respondents were excluded because of missing data. Finally, 499 respondents were included in this study ( Figure 1).

### Instrument

Participants completed a structured self-administered questionnaire, which was subsequently uploaded to an online survey software (Google Forms), as well as an instant messaging application (WhatsApp). The objective of the study, consent information to assure voluntary participation, as well as participants’ anonymity and confidentiality, were all included in the introduction section of the questionnaire. The questionnaire was easily accessible to the participants. Their responses were exported and analyzed once collected. The 17-question questionnaire yielded the following results: (a) Demographic characteristics, (b) Work or occupation, (c) Exercises performed prior to COVID-19 infection (type, frequency and duration), (d) Daily habits and tasks, (e) Chronic disease complaints (high blood pressure, heart and lung diseases, diabetes, cancer), (f) Duration of COVID-19 infection, (g) Accompanied Symptoms during COVID-19 infection (whether the symptoms persisted after recovery, duration and the target symptoms persisted). All the questions were written in basic, brief, and straightforward language, and the responses were organized in a straightforward manner.

### Data collection

The responses were gathered using a Google form. The survey link was sent to potential participants via WhatsApp and email. To increase the response rate, two reminders were sent at three-days interval and the responses were organized in a Microsoft Excel sheet 2010.

### Statistical analysis

The results were analyzed with SPSS version 24 (SPSS Inc, Chicago, Illinois, USA) (RRID:SCR_002865). Chi-squared test was performed to calculate the percentages and correlations between categorical variables in the descriptive analysis. Percentages with a 95% confidence interval were also computed. The significance level was established at a *p*-value of 0.05. ANOVA test was utilized to compare the disability scores among participants. Since the duration of long-term symptoms was skewed, the median and interquartile range (IQR) were chosen to summarize the data. For categorical and ordinal variables, proportion was used.

## Results

A total of 499 responses were presented and analyzed. Duration of persistent symptoms was set from one month to three months. Females made up nearly 70% of the samples, with the majority aged under 45 years. Among the participants, 245 (49.1%) were employed, including 60 (24.9%) civilian workers, 38 (15.8%) military members, 37 (15.4%) medical workers, and 254 (50.9%) were unemployed. Out of 499 respondents, 275 (55.1%) of the participants reported that they had practice of exercising before COVID-19 infection (mostly walking, 67.6%), while 224 (44.9%) had not participated in any type of exercises before COVID-19 infection. 394 (69.9%) females were affected, while 150 (30.1%) men were affected. Most of them (420 = 84.2%) were under 45 years old, and 79 (15.8%) were over 45 years old. 405 (81.2%) of the participants came from the Western region of Saudi Arabia. About 118 (23.6%) of the participants had chronic diseases, including respiratory disease (42 = 8.4%), diabetes mellitus (DM) (41 = 8.2%), hypertension (HT) (26 = 5.2%), cancer (5 = 1.0%), and cardiac disorders (3 = 0.6%). 381 (76.4%) participants were free of chronic diseases ( Table 1).

**Table 1.** Participants demographic characteristics (N = 499).

Variables		Frequency (n)	Percentage (%)
Gender	Female	150	30.1
	Male	349	69.9
Age	Less than 45 years	420	84.2
	45 years or above	79	15.8
Region	Western region	405	81.2
	Other regions	94	18.8
Currently employed		245	49.1
Has no chronic diseases		381	76.4
Has chronic disease		118	23.6
Participants practicing exercises before COVID-19 infection		275	55.1
Participants did not practice any exercises before COVID-19 infection		224	44.9
Chronic diseases		118	23.6
Respiratory diseases		42	8.4
Cardiac diseases		3	0.6
Cancer		5	1.0
Diabetes miletus		41	8.2
Hypertension		26	5.2
Others		20	4.0

In this study, headache (63.1%) was the most reported symptom after COVID-19 infection, followed by muscle ache or weakness (62.3%), vertigo (25%), concentration problems (21.8%), breathing troubles (20.4%), and loss of balance (19.4%). On the other hand, five cases of seizure (1%, four cases of intermittent seizure and one case of continuous seizure), and three cases of Guillain-Barre Syndrome (0.6%) have been documented and were the least common symptoms. Many participants were suffering from more than one persistent symptoms after COVID-19 infection, so the percentages were more than 100 percent ( Table 2).

**Table 2.** Persistent symptoms after COVID-19 infection.

Symptoms	Frequency (N = 499)	Percentage (%)
Headache	315/499	63.1
Muscle pain	310/499	62.3
Vertigo	125/499	25.0
Concentration problems	109/499	21.8
Loss of balance	97/499	19.4
Seizure	5/499	1.0
Guillain Barre Syndrome	3/499	0.6

Certain symptoms, such as breathing problems and muscle discomfort or weakness persisted after COVID-19 infection had cleared up in a median duration of 30 days and an interquartile range of 45 days. Regarding muscle pain, back muscles were the most affected (122 = 24.4%), followed by lower limb muscles (77 = 15.4%), neck muscles (30 = 6.0%), pelvic muscles (19 = 3.8%), and upper limb muscles (17 = 3.4%) ( Table 3).

**Table 3.** Muscle pain or weakness.

Muscular pain	Frequency (N = 499)	Percentage (%)
Neck muscles	30	6.0
Back muscles	122	24.4
Upper limb muscles	17	3.4
Lower limb muscles	77	15.4
Pelvic muscles	19	3.8
Other	234	46.9

Many participants in this study showed a link between reported symptoms and certain physical activity performance after COVID-19 infection. Gender and physical activity performance were shown to have a significant relationship in 146 (29.3%) of participants ( *p* = .001). There were 118 (80.8%) females and 28 (19.2%) males. There was a significant link between persistent symptoms’ impact on physical activity performance and respiratory disease ( *p* = 0.017), with 330 (93.5) patients without respiratory diseases recording no persistent symptoms on physical performance and 127 (87.0) patients with respiratory diseases recording persistent symptoms on physical performance. No significant association were found between physical activity performance after COVID-19 infection and the habit of exercising before COVID-19 infection. 80 (54.8%) of the subjects had habit of exercising before the infection, whereas 66 (45.2%) had not. Furthermore, 195 (55.2%) had habit of exercising before the infection, which had no effect on their physical activity performances, while 158 (44.8%) had no habit of exercising before the infection and had no complaints of physical performance performances.

A majority of 353 participants (70.7%) do not record any association between persistent symptoms and certain physical activity performance, 231(65.4%) were females and 122 (34.6%) were males. Patients without hypertension had the highest comorbidity value of 337 (95.5%), followed by those without respiratory diseases (330 = 93.5%), non-diabetic patients (323 = 91.5%), diabetic patients (30 = 8.5%), patients with respiratory diseases (23 = 6.3%), and patients with hypertension (16 = 4.5%) ( Table 4).

**Table 4.** Association between the impact of symptoms, physical activity and demographic characteristics (N = 499).

Factor		Yes n (%)	No n (%)	*p*-value
Gender		146 (29.3)	353 (70.7)	
	Male	28 (19.2)	122 (34.6)	0.001
	Female	118 (80.8)	231 (65.4)	
Age	Less than 45 y/o	117 (80.1)	303 (85.8)	0.113
	45 y/o or older	29 (19.9)	50 (14.2)	
Exercise before COVID-19 infection	Yes	80 (54.8)	195 (55.2)	0.927
	No	66 (45.2)	158 (44.8)	
Comorbidities	Respiratory disease	19 (13.0)	23 (6.5)	0.017
	Non-respiratory disease	127 (87.0)	330 (93.5)	
	DM	11 (7.5)	30 (8.5)	0.019
	Non-DM	135 (92.5)	323 (91.5)	
	HT	10 (6.8)	16 (4.5)	0.012
	Non-HT	136 (93.2)	337 (95.5)	

*p*-value attained by chi-square test, DM: Diabetes mellitus, HT: Hypertension.

Additionally, participants who recorded persistent symptoms were asked to specify the frequency of symptoms. However, significant association was found between gender and frequency of symptoms (from 58 participants who recorded symptoms to be always persisting); females (47 = 81%) recorded higher frequency than males (11 = 19%), as shown in Table 5. Another significant association was discovered between respiratory disorders before infection and the prevalence of persistent symptoms, ( *p* < 0.001). The findings showed that 280 (98.2%) of those who had no respiratory disorders before COVID-19 infections had no symptoms after the infection, while 5 (1.8%) of those who had respiratory diseases before COVID-19 infection had no symptoms after the infection.

**Table 5.** Frequency of symptoms affecting daily activity and demographic variables.

Factor		Always n (%)	Sometimes n (%)	Never n (%)	*p*-value
N = 499		58 (11.6)	303 (60.7)	138 (27.7)	
Gender	Male	11 (19)	87 (28.7)	52 (37.7)	0.024
	Female	47 (81)	216 (71.3)	86 (62.3)	
Age	Less than 45 y/o	50 (86.2)	247 (81.5)	123 (89.1)	0.115
	45 y/o or older	8 (13.8)	56 (18.5)	15 (10.9)	
Exercise before COVID-19 infection	Yes	31 (53.4)	164 (54.1)	80 (58.0)	0.726
	No	27 (46.6)	139 (45.9)	58 (42.0)	
Comorbidities	Respiratory disease	14 (24.1)	23 (14.7)	5 (1.8)	<0.001
	Non-respiratory disease	44 (75.9)	133 (85.3)	280 (98.2)	

*p*-value attained by chi-square test, DM: Diabetes mellitus, HT: Hypertension.

Table 5 provided more information about the participants who reported the presence of symptoms occasionally while performing everyday activities, 133 (85.3%) without respiratory diseases and 23 (14.7%) with respiratory diseases. Other demographic data and comorbidities failed to reveal any statistically significant association between the impact and physical activity (age, practicing exercises before infection).

## Discussion

To our knowledge, this is the first study to evaluate the prevalence of musculoskeletal, neurological and physical disorder after COVID-19 in the KSA adult population and their relationship with physical activities. The purpose of the current study is to determine the prevalence of musculoskeletal, neurological, and physical health problems after COVID-19 infection, as well as how it affects the capability of daily life activities. Overall, this study found that musculoskeletal, neurological, and physical activity were negatively affected after COVID-19 infection. This study involved 499 participants. According to demographic data, the percentage of women infected COVID-19 (349 = 69.9%) was higher than that of men (150 = 30.1%). This is different from a previous study in the KSA, in which the authors reported a higher prevalence among males than females. This is agreed with the percentage of total daily cases reported in the same study, in which male was decreased by 18.0%, compared to a 150% increase in females. ^8^ Participants under 45 years old were also more impacted (420 = 84.2%) than those over 45 years old (79 = 15.8%), which matched the findings of a pervious study (Alyami *et al.*, 2020) that COVID-19 was more prevalent in adults than in children and elderlies. Our findings revealed that 275 persons (55.1%) had practiced physical activities before COVID-19 infection, which is higher than that reported by the General Authority for Statistics in 2019. ^9^ This could be due to the larger sample size and increased awareness of the health benefits of physical activity among the Saudi population.

Low back pain was the most frequently recorded symptom among the participants after COVID-19 infection. The results are similar to those reported in a cross-sectional study conducted in Riyadh, Saudi Arabia with a sample of 463 participants. The reason for this is most likely owing to the detrimental impacts of quarantine for most nations during the Corona epidemic, as stated in a cross-sectional study done in Riyadh, Saudi Arabia with a sample size of 463 people. Sitting for long periods during quarantine may reduce lumbar muscle activity, which overloads the body’s passive tissues, such as intervertebral discs and ligaments. ^10^ Furthermore, pain in the lower limb muscles was identified as the second most prevalent muscle pain in this study, which coincided with the previous study and could be due to the same reasons that produce low back pain. ^10^ The presence of musculoskeletal manifestations in the current study findings could be explained by the fact that the Saudi Arabian government’s quarantine decree, as well as its strict implementation to ensure the preservation of public health for all people and the commitment of all people to it, which influenced the population’s lifestyle in terms of physical activity, dietary habits, and mental health. This phenomenon contributed to musculoskeletal problems, supported by multiple studies showing that physical activity rates were dramatically reduced, and food habits were negatively impacted following the lockdown. ^11^ The high prevalence of muscle discomfort, particularly low back pain and other musculoskeletal complaints, may be explained by long hours of screen usage for work and education.

In this study, the impacts of COVID-19 on physical activity were recorded, and certain parameters such as gender and respiratory disease were found to be significantly related. The proportion of females who reported an impact on physical activity was 33.8%, compared to 18.67% of males ( *i.e.*, participants who declared that COVID-19 infection had a negative impact on their physical activity), like another finding, in which patient’s walking time was significantly reduced six months after the onset of persistent symptoms after COVID-19 infection. ^12^ It could also be due to the muscle mass in male bodies and the fact that males, on average, engage in more physical exercise than females. Another viewpoint is that females are more likely to babysit and care for their children’s schooling, which has unquestionably altered substantially and become a bigger burden during COVID-19 pandemic, owing to quarantine and social isolation.

COVID-19 infection had a greater influence on physical activity among respondents with respiratory disease, which could lead to a negative impact on patients’ physical activity and cause further difficulties. However, more research is needed to confirm this link. Most people become sedentary as a result of the shift in lifestyle and the transformation of work and education to teleworking and tele-education. Impacts of COVID-19 was thoroughly documented in literature both locally and globally. ^11^^,^^12^ Physical activity was found to be strongly affected by quarantine and course of disease in a systematic review of 15 publications. ^11^ Furthermore, the reduction in physical activity after COVID-19 has a severe impact on people’s mental health and could lead to a variety of psychiatric problems. ^13^

During the COVID-19 pandemic, the prevalence of physical activity among participants was drastically reduced. Both active and inactive people became less active than they were before the lockdown. ^14^^,^^15^ In Saudi Arabia, a survey-based study with 2,255 participants found comparable results, with more than half of respondents reporting a drop-in physical activity. ^10^ However, the current study found a more significant link between COVID-19 infection and persistent symptoms in diabetic patients than non-diabetic patients, and in hypertension patients than patients without hypertension, which is supported by another study that found a higher prevalence of comorbidities with DM (68.3%), and hypertension (42.6%) after COVID-19 infection. ^16^ As a result, patients with diabetes and chronic hypertension should be cautious about the long-term effects of COVID-19 infection on physical activity and muscular soreness after recovery.

## Conclusion

COVID-19 had impacts on respiration, muscle strength, and daily activities. After COVID-19 infection, muscle ache and headache persisted in higher proportions than the other symptoms. Capability of daily activities was correlated to gender, respiratory diseases, diabetes, and hypertension.

### Recommendation

Cohort studies with sufficient follow-up duration should be performed for more informative and precise results. Larger sample size will be needed in future studies for more generalized results.

## Data availability

### Underlying data

Figshare: Underlying data for ‘Prevalence of Musculoskeletal, Neurological and Physical Disorder After COVID-19 in Saudi Arabia: A Cross Sectional Study

The project contains the following underlying data:

Flow diagram of sample selection: 10.6084/m9.figshare.18585995

Tables: 10.6084/m9.figshare.18586016

Raw data: 10.6084/m9.figshare.18586034

STROBE-checklist for observational study: 10.6084/m9.figshare.19161485

### Extended data

The project contains the following extended data:

Questionnaire: 10.6084/m9.figshare.19161620

Data are available under the terms of the Creative Commons Zero “No rights reserved” data waiver (CC0 1.0 Public domain dedication)
