# Supplementary material for: Prevalence of Musculoskeletal, Neurological and Physical Disorder After COVID-19 in Saudi Arabia: A Cross Sectional Study
Source: F1000Res. 2023 Sep 13;11:463. Originally published 2022 Apr 26. [Version 3] doi: 10.12688/f1000research.109007.3 (PMC11401979; doi:10.12688/f1000research.109007.3)
Supplement: Peer review history

# Reviewer report: Reviewer response for version 3

**DOI:** 10.5256/f1000research.155834.r206197

**Published:** 2024-09-14

**Authors:** Camelia Pescaru

**Affiliations:** 1 University of Medicine, Timișoara, Romania

No further comments to make.

Is the work clearly and accurately presented and does it cite the current literature?

Partly

If applicable, is the statistical analysis and its interpretation appropriate?

Yes

Are all the source data underlying the results available to ensure full reproducibility?

Partly

Is the study design appropriate and is the work technically sound?

Yes

Are the conclusions drawn adequately supported by the results?

Yes

Are sufficient details of methods and analysis provided to allow replication by others?

Yes

Reviewer Expertise:

Areas of expertise: acute and chronic pulmonary diseases, such as COPD, Asthma, Sarcoidosis, Covid-19, Cystic fibrosis.

I confirm that I have read this submission and believe that I have an appropriate level of expertise to confirm that it is of an acceptable scientific standard.

# Reviewer report: Reviewer response for version 2

**DOI:** 10.5256/f1000research.137299.r196018

**Published:** 2023-09-04

**Authors:** Camelia Pescaru

**Affiliations:** 1 University of Medicine, Timișoara, Romania

In figure 1 it states that 2000 people were screened for eligible participation, however in the sampling part of the article, that number isn't referred to, nor does it say why 800 people were excluded, therefore it should mention the criteria of exclusion that leads to the 1200 people that were randomly approached. If this was supposed to be understood from the Random selection of participants from different district in KSA, then also mention this number there and insert another box adjacent to it with the exclusion criteria (if it was randomly selected by a program) just like the other text boxes below.

 Regarding citing the current literature, 15 of the 16 references are from the last 3-4 years, while reference number 3 is from 10 years ago, try looking for an alternative that is in the 2019-2022 range so that it matches with the rest of the references.

Is the work clearly and accurately presented and does it cite the current literature?

Partly

If applicable, is the statistical analysis and its interpretation appropriate?

Yes

Are all the source data underlying the results available to ensure full reproducibility?

Partly

Is the study design appropriate and is the work technically sound?

Yes

Are the conclusions drawn adequately supported by the results?

Yes

Are sufficient details of methods and analysis provided to allow replication by others?

Yes

Reviewer Expertise:

Areas of expertise: acute and chronic pulmonary diseases, such as COPD, Asthma, Sarcoidosis, Covid-19, Cystic fibrosis.

I confirm that I have read this submission and believe that I have an appropriate level of expertise to confirm that it is of an acceptable scientific standard, however I have significant reservations, as outlined above.

# Response

**Published:** 2023-09-08

**Authors:** Anwar Ebid

**Affiliations:** Umm-Al-Qura University, Saudi Arabia

Thank you for taking the time to review our submission, and for your valuable comments and nice words. Please find our detailed response to your comments below. All text in the manuscript has been added or changed.

 Authors reply to Comment # 1

 The methodology part has been revised, and the sampling section has been completely rewritten to describe the two stages of sample selection as the following:

- On a list of patients in the hospital database, 2000 participants were randomly selected for the first sample stage; however, 800 people had to be eliminated because of contact technical issues. The second stage involved approaching 1200 participants, but only 520 patients returned the questionnaire and 680 declined to participate. 21 respondents were excluded due to missing data. Finally, this study included 499 respondents.

- The updated sample selection flow diagram now includes the participant exclusion criteria.

Authors reply to Comment # 2

 Reference No. 3 was replaced by a recent one (2022)

- Romero CNS; Colen HI; Godoy RME et al.: Clinical signs and symptoms associated with COVID-19: a cross-sectional study *. Int. J. Odontostomat., 16(1)*:112-119, 2022.

# Reviewer report: Reviewer response for version 1

**DOI:** 10.5256/f1000research.120457.r143364

**Published:** 2022-08-01

**Authors:** Salah Abd Elmonem Sawan

**Affiliations:** 1 Department of Physical Therapy for Neuromuscular Disorder and its Surgery, Faculty of Physical Therapy, Cairo University, Giza, Egypt

The paper is valuable and it is clear, simple and with good sound but I have some questions and I need the answers from the authors:

**1. How could they assure that the link they sent for the patients was only for Saudi people? **i.e., Is it possible to provide the reader with a simple explanation of how to send the query to Saudis only, that is, how can you ensure that the link sent was answered by Saudis only because you made this query for Saudis only?

**2.** **How was the query carried out, and how were the questions added?** Have you hired a specialist to review the questions about the questionnaire? Was it approved and reviewed by a specialized authority? If you have, please add this to the paper in methodology section.

**3. Was the survey (Questionnaire) provided in Arabic language or in another language?**

**4. Are there any further symptoms** **following** **COVID-19 were not mentioned at the paper?**

**5. Were there another symptoms specific to people who have been vaccinated against the Corona virus at this work?**

 Thank you. 

 Dr Salah Sawan

Is the work clearly and accurately presented and does it cite the current literature?

Yes

If applicable, is the statistical analysis and its interpretation appropriate?

Yes

Are all the source data underlying the results available to ensure full reproducibility?

Yes

Is the study design appropriate and is the work technically sound?

Yes

Are the conclusions drawn adequately supported by the results?

Yes

Are sufficient details of methods and analysis provided to allow replication by others?

Yes

Reviewer Expertise:

Physical therapy and movements disorders

I confirm that I have read this submission and believe that I have an appropriate level of expertise to confirm that it is of an acceptable scientific standard.

# Response

**Published:** 2022-08-09

**Authors:** Anwar Ebid

**Affiliations:** Umm-Al-Qura University, Saudi Arabia

Dear Professor, Salah Sawan

 Thank you so much for your comments.

 The authors are grateful for the reviewers' voluntary contributions in the form of insightful comments that have assisted us in improving our manuscript.

 The authors have made every effort to revise the manuscript in response to your comments.

 In response to your suggestions, we have published an updated version of this article.

 Please consider these new versions to be the revised version of this article.

 Thank you very much.

 Regards,

 Authors

 The paper is valuable and it is clear, simple, and with good sound but I have some questions and I need the answers from the authors:

 1. How could they assure that the link they sent for the patients was only for Saudi people? i.e., Is it possible to provide the reader with a simple explanation of how to send the query to Saudis only, that is, how can you ensure that the link sent was answered by Saudis only because you made this query for Saudis only?

**Reply: The King Abdullah International Medical Research Center (KAIMRC) was in responsible of selecting Saudi participants at random using a filing system and distributing the questionnaire via WhatsApp.**

 2. How was the query carried out, and how were the questions added? Have you hired a specialist to review the questions about the questionnaire? Was it approved and reviewed by a specialized authority? If you have, please add this to the paper in the methodology section.

**Reply: The research team first prepared the query, which was then sent to two different Arabic specialists to modify and correct any Arabic errors before being sent to a documented English translation office to prepare the final version of the questionnaire. It was reviewed by the ethical committee in The King Abdullah International Medical Research Center (KAIMRC). The questionnaire was submitted as a preliminary sample to see if there were any issues with it. The response was added to the methodology section.**

 3. Was the survey (Questionnaire) provided in Arabic language or in another language?

**Reply: Yes: The survey was provided in Arabic and English version after reviewing by specialists, also it was reviewed by the ethical committee in The King Abdullah International Medical Research Center (KAIMRC)**

 4. Are there any further symptoms following COVID-19 were not mentioned at the paper?

**Reply: All symptoms following COVID-19 mentioned with the respondent were reported in the paper**

 5. Were there another symptoms specific to people who have been vaccinated against the Coronavirus at this work?

**Reply No:  There were no symptoms specific to people who had been vaccinated against the Coronavirus at this work.**

**The questionnaire was only used to assess and collect symptoms after Covid-19, and we did not test for vaccine effect.**
